# HSP104 and HSP20‐L Are Required by *Aspergillus nidulans* in Response to Attack by Fungivorous Springtail *Sinella curviseta*

**DOI:** 10.1111/1758-2229.70147

**Published:** 2025-07-06

**Authors:** Xiaomeng Wang, Juan Xi, Pengxu Chen, Yingying Chen, Keyu Chen, Weifa Zheng, Yanxia Zhao

**Affiliations:** ^1^ School of Life Sciences Jiangsu Normal University Xuzhou China

**Keywords:** *Aspergillus nidulans*, biotic stress, cell wall, heat shock protein, spores

## Abstract

Heat shock proteins (HSPs) are conserved biomolecules that are consistently expressed and upregulated in response to stress. However, whether fungi activate HSPs in response to fungivorous arthropods' attack remains unclear. In this study, we investigated the function of HSP104 and HSP20‐L in *Aspergillus nidulans* upon *Sinella curviseta* stress. The results revealed that *hsp*104 and *hsp*20‐L were upregulated upon the stress. Knockout of *hsp*104 and/or *hsp*20‐L inhibited conidia and cleistothecia formation. Additionally, *S. curviseta* stress inhibited conidia and cleistothecia formation in the wild‐type strain. *hsp*104 positively regulated conidia formation in response to stress, while *hsp*20‐L negatively regulated it. Notably, *hsp*104 and *hsp*20‐L exhibited opposing functions on ascospore formation upon biotic stress. The absence of *hsp*104 and/or *hsp*20‐L and *S. curviseta* stress resulted in increased cellular damage. During asexual development, both *hsp*104 and *hsp*20‐L promoted chitin and β‐glucan synthesis and catalase activity. During sexual development, only chitin synthesis was enhanced in Δ*hsp*104 and Δ*hsp*20‐L. Under *S. curviseta* stress, HSP104 promoted chitin synthesis and catalase activity during asexual development, whereas HSP20‐L promoted chitin and trehalose synthesis and superoxide dismutase activity during sexual development. Collectively, our results suggest that *hsp*104 and *hsp*20‐L play a role in response to *S. curviseta* stress to maintain homeostasis.

## Graphical abstract

The heat shock proteins HSP104 and HSP20‐L of *A. nidulans* exhibit a reciprocal regulatory relationship to maintain physiological homeostasis in response to *S. curviseta* ‐induced stress.

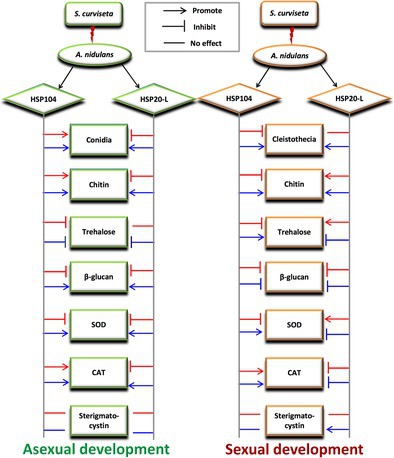

## 1 Introduction

Widely distributed filamentous fungi play an important role in ecosystems. During growth and development, they encounter various abiotic and biotic stresses. Abiotic stresses primarily arise from factors such as temperature fluctuations, pH, and exposure to heavy metals, while biotic stresses originate from pathogens, insects, and nematodes (Steinberg 2012). Fungi adapt to or defend against unfavourable environments by regulating their morphology and metabolic processes.

Filamentous fungi develop hyphae and spores that can serve as food sources for insects and nematodes, thereby posing a threat to their growth. When *Neurospora crassa* is subjected to fungivorous arthropod stress, it mitigates damage by altering its structure along with regulating growth and metabolism (Zhao et al. 2017; Sun et al. 2023; Lu et al. 2024). *Aspergillus fumigatus* employs the cAMP signalling pathway to modulate its morphology and asexual reproduction during interaction with arthropods (Silva et al. 2020). In addition, *A. fumigatus* not only encapsulates ascospore within cleistothecia but also enriches the melanin content of the spore wall layer, thereby improving spore viability and environmental adaptability (Calvo et al. 2002). Various forms of stress can prompt fungi to activate defence mechanisms. Filamentous fungi modify the composition of their metabolome in response to biotic stresses (Sun et al. 2023). Different species exhibit distinct defending strategies when confronted with invasion by diverse organisms.

*Aspergillus* is widely distributed in nature, and *Aspergillus nidulans* serves as a model fungus due to its fast growth and convenient genetic manipulation. *A. nidulans* produces conidia through asexual development while forming cleistothecia containing ascospores via sexual development (Dyer and O'Gorman 2011; Etxebeste et al. 2010). It is commonly found in food, soil, and air. Moreover, it produces mycotoxins that pose risks to human health and environmental safety (Caceres et al. 2020). Consequently, identifying effective strategies for inhibiting mould growth remains a challenge.

Stress‐induced alterations in gene expression are central to the adaptive responses of fungi to harsh environments (Gasch 2007; Gasch et al. 2000). Heat shock proteins (HSPs) are a class of proteins that can engage in various cellular functions in fungi (Roy and Tamuli 2022). Studies have indicated that HSPs play a role in response to a variety of environmental stresses, including pH, temperature, starvation, heavy metal, osmotic stress, and oxidative stress (Tiwari et al. 2015; Kregel 2002). HSPs are constitutive and stress‐inducible components that are present in almost all organisms (Tiwari et al. 2015).

HSPs are classified into several families such as HSP100, HSP90, HSP70, HSP60, HSP40, and small HSPs (sHSPs) (Tiwari et al. 2015; Kregel 2002). The expression of Hsp104 is observed under high‐pressure conditions (Sanchez et al. 1992). HSP90 functions as a dimeric ATPase crucial for fungal growth, cell survival, and pathogenicity (Rocha et al. 2021; Kregel 2002). Both HSP70 and sHSPs are involved in fungal development, tolerance to stress conditions, and drug resistance (Tiwari et al. 2015; Kregel 2002; Yu et al. 2015). Additionally, HSP60 serves as a mitochondrial chaperone protein (Verghese et al. 2012), and HSP40 regulates the ATPase machinery in fungi (Malinverni et al. 2017). sHSPs play multiple roles in fungal stress response, developmental processes, and drug resistance (Welker et al. 2010). In *A. nidulans* , sHSPs are involved in heat, cold, osmotic, and oxidative stress resistance (Wu et al. 2016). HSP104 is associated with heat tolerance, survival, and ethanol tolerance (Glover and Lindquist 1998; Boreham and Mitchel 1994). HSP21 plays a role in fungal adaptation to environmental stress and pathogenicity by regulating glycerol and glycogen, while also promoting hyphal formation (Mayer et al. 2012; Lopez‐Matas et al. 2004). Furthermore, salt and oxidative stress, as well as hot and cold conditions, lead to an increase in the expression of *hsp20*‐L in *A. nidulans* (Wu et al. 2016), and the transcription of *hsp*30, *hsp*70, and *hsp*90 is regulated by PalA in response to acid pH sensing (Freitas et al. 2011). HSPs, as molecular chaperone proteins, participate in a variety of physiological functions in *A. nidulans* (Freitas et al. 2011). Although HSPs play a role in response to temperature (Matsushita et al. 2009), osmotic pressure, pH (Freitas et al. 2011), and other abiotic stresses, the function of HSPs in response to biotic stresses is unknown.

*Sinella* represents a group of microarthropods that mainly feed on fungi, humus, and lichens (Rohlfs et al. 2011). *S. curviseta* is a widely distributed springtail found in the natural environment and feeds on filamentous fungi. *A. nidulans* is a typical filamentous fungus that forms mycelia, produces conidia during asexual reproduction, and forms cleistothecia during sexual development. Therefore, we established the *A. nidulans* – *S. curviseta* model to investigate how filamentous fungi respond to the fungivores attack by HSPs.

*A. nidulans* may respond to biotic stress through dramatic and comprehensive changes in gene expression patterns, enzyme activity alterations, and metabolism adjustments. In this study, we aimed to investigate the mechanisms by which *A. nidulans* responds to *S. curviseta* stress by regulating growth and development, cell wall composition, and antioxidant enzyme activities through HSPs.

## 2 Experimental Procedures

### 2.1 Strains and Culture Conditions

*A. nidulans* strains utilised in this study are described in Table S1. All strains were cultured in glucose minimal medium (GMM) and sexual medium (SM) as previously described by Ni and Yu (2007). For the cultivation of *pyrG−auxotrophic* strains, the medium was supplemented with uridine and uracil. In the case of *pyroA−auxotrophic* strains, the medium was supplemented with pyridoxine hydrochloride. For *S. curviseta* stress, the cultures were incubated at 37°C for 3 days and then subjected to attack with 20 *S. curviseta* individuals for 3 days.

### 2.2 Mutants Construction

Briefly, *hsp* deletion mutants were constructed through homologous recombination. The primer sequences are provided in Table S2. *A. nidulans* RJMP1.59, which is auxotrophic for uracil/uridine and pyridoxine hydrochloride, served as the recipient strain. The 5′ and 3′ flanking regions of *hsp*104 and *hsp*20‐L were amplified from genomic DNA of *A. nidulans* FGSC4. Additionally, the *pyrG* gene was amplified from genomic DNA of *A. fumigatus* (AF293). The fusion‐PCR constructs were generated using nested primers. The deletion cassettes were subsequently introduced onto *A. nidulans* RJMP1.59 protoplasts. To generate Δ*hsp*104::Δ*hsp*20‐L mutant, the *hsp*20‐L gene was deleted from Δ*hsp*104 genome using *pyroA* as a selection marker. All mutants were verified by PCR. At least three independent deletion strains were isolated and confirmed.

To generate *hsp* overexpression strains, the *hsp* genes were amplified from the genomic DNA of *A. nidulans* FGSC4. Overexpression strains of the target genes were constructed using plasmid pHS11 harbouring a nitrogen‐inducible *niiA* promoter. The overexpression strains were confirmed by Semi‐Quantitative RT‐PCR. At least three independent overexpression strains were isolated and confirmed. The modified GMM containing nitrogen sources was used to induce the promoter.

### 2.3 Response of *hsp* Mutants to *S. curviseta* Stress

The response of the strains to *S. curviseta* stress was assessed according to Sun et al. (Sun et al. 2023). For asexual development, 1 × 10^7^ conidia of each strain were inoculated onto solid GMM and incubated at 37°C for 3 days. Then, approximately 20 *S. curviseta* individuals were released onto the plate. Subsequently, the cultures were further incubated at room temperature for an additional 3 days. Conidia were harvested using Tween 20 and counted with a haemocytometer.

For sexual development, 1 × 10^7^ conidia from each strain were inoculated onto solid SM, incubated at 37°C for 6 days, and then 20 *S. curviseta* individuals were released onto plates. Subsequently, the cultures were incubated at room temperature for 6 days. The formation of cleistothecia was observed using a microscope (Motic M150). All experiments were conducted in triplicate.

### 2.4 *hsp*104 and *hsp*20‐L Expression Levels in Different Strains

A suspension containing 1 × 10^7^ *A. nidulans* spores was dispersed and inoculated onto solid GMM for 3 days at 37°C. The cultures without insect stress served as controls. After 3 days of incubation at room temperature, the cultures were collected using a spatula and frozen with liquid nitrogen. RNA was extracted using Trizol and the expression was quantitatively analysed by reverse transcription‐polymerase chain reaction (RT‐PCR). The assays were carried out with the primers listed in Table S2. The actin gene was used as a reference. The procedures involving RNA extraction, cDNA synthesis, and RT‐PCR assay were performed as described previously (Zhao et al. 2021). The expression changes were calculated by the 2C_t_ analytic method. Results are the means (standard deviations) of three triplicate assays.

*Hsp* expression was measured under *S. curviseta* stress and compared to the expression levels in the wild‐type strain. To investigate the relationship between *hsp*104 and *hsp*20‐L, their expressions were evaluated in the wild‐type strain and *S. curviseta* ‐induced strains. The expression of *hsp*104 was measured in Δ*hsp*20‐L, and that of *hsp*20‐L in Δ*hsp*104.

### 2.5 Propidium Iodide Staining

Conidia were collected and the propidium iodide (PI) solution was added to achieve a final concentration of 3 μmol/L. The samples were rinsed twice at 37°C and the fluorescence intensity was measured by a microplate reader (Synergy H4 Hybrid Reader) (Niu et al. 2024).

### 2.6 Extraction and Quantification of Chitin

The cultures of different strains were collected and soaked in concentrated hydrochloric acid for 24 h. Subsequently, the pH was adjusted to 7.0 with sodium hydroxide. Next, acetylacetone was added, and the mixture was carried out at 90°C for 1 h. Anhydrous ethanol and *p*‐dimethylaminobenzaldehyde were added, and the reaction was continued for 1 h at room temperature. The absorption value of glucosamine, the hydrolysate of chitin at 530 nm, was detected by a spectrophotometer (Lehmann and White 1975; Zhang et al. 2017).

### 2.7 Extraction and Quantification of Trehalose

The cultures were ground with liquid nitrogen before adding distilled water at 80°C. Then, the mixture was shaken for 15 min. The supernatant obtained after centrifugation at 10000 rpm for 3 min was the crude extract of trehalose. The content of trehalose was detected using a trehalose kit (Megazyme, Ireland, product No. K‐TREH).

### 2.8 Extraction and Quantification of β‐Glucan

Cultures of wild‐type and mutant strains with or without *S. curviseta* stress were collected. β‐glucan content was measured via the aniline blue assay method as described by Fortwendel et al. (2008). Fluorescence measurement was performed using a microplate reader with an excitation wavelength of 398 nm and an emission wavelength of 502 nm. The standard curve was generated based on dilutions of β‐glucan.

### 2.9 Determination of Superoxide Dismutase and Catalase Activities

Grind the collected cultures with liquid nitrogen. Add an equal volume of Hepes buffer (containing PMSF) to the milled samples, mix, and then ice bath for 10 min. The procedure was repeated three times. Then centrifuge the samples at 12000 rpm for 15 min at 4°C. The supernatant is the crude protein extract. The protein concentration was measured by the Coomassie Brilliant Blue method (Nanjing Jiancheng Biotechnology Co. LTD., item No. A045‐2‐2). The activity of superoxide dismutase (SOD) and catalase (CAT) was detected by SOD (Nanjing Jiancheng Biotechnology Co. LTD., item No. A001‐1‐2) and CAT (Nanjing Jiancheng Biotechnology Co. LTD., item No. A007‐1‐1) kits respectively.

### 2.10 Extraction and Quantification of Sterigmatocystin

Cultures of different strains with or without *S. curviseta* stress were collected. Sterigmatocystin was extracted using an ultrasonic crusher with trichloromethane as the extraction solvent. The quantification of sterigmatocystin was determined via HPLC. The mobile phases utilised included: (1) an aqueous solution containing 5% acetonitrile and 0.05% formic acid, and (2) an acetonitrile solution containing 0.05% formic acid (Park et al. 2017).

### 2.11 Non‐Targeted Metabolomics Testing

The cultures of wild‐type and mutant strains with or without *S. curviseta* stress were collected and mixed after adding the extraction solution (methanol‐acetonitrile‐water, V/V = 2:2:1). Next, steel beads were added to the mixture and processed on a 45 Hz grinder for 10 min, followed by sonication in an ice bath for 10 min, and finally the extract was concentrated using a rotary evaporator. The LC/MS system (Waters Acquity I‐Class PLUS ultra‐high performance liquid tandem Waters Xevo G2‐XS QT of high resolution mass spectrometer) was used to detect the metabolite composition. MassLynx V4.2 was utilised to collect raw data, and data processing such as peak extraction and peak alignment were performed by Progenesis QI software. Identification was carried out based on the online METLIN database, the public database, and Biomarker Technologies self‐built library using Progenesis QI software, while theoretical fragment identification was performed. The mass deviation of parent ions was within 100 ppm, and the mass deviation of fragment ions was within 50 ppm.

### 2.12 Statistical Tests

Statistical differences were assessed using SPSS 20.0. Data are expressed as mean ± standard deviation (SD). The *p* value < 0.05 was considered statistically significant.

## 3 Results

### 3.1 Expression Levels of *hsp*104 and *hsp*20‐L

**FIGURE 1:**
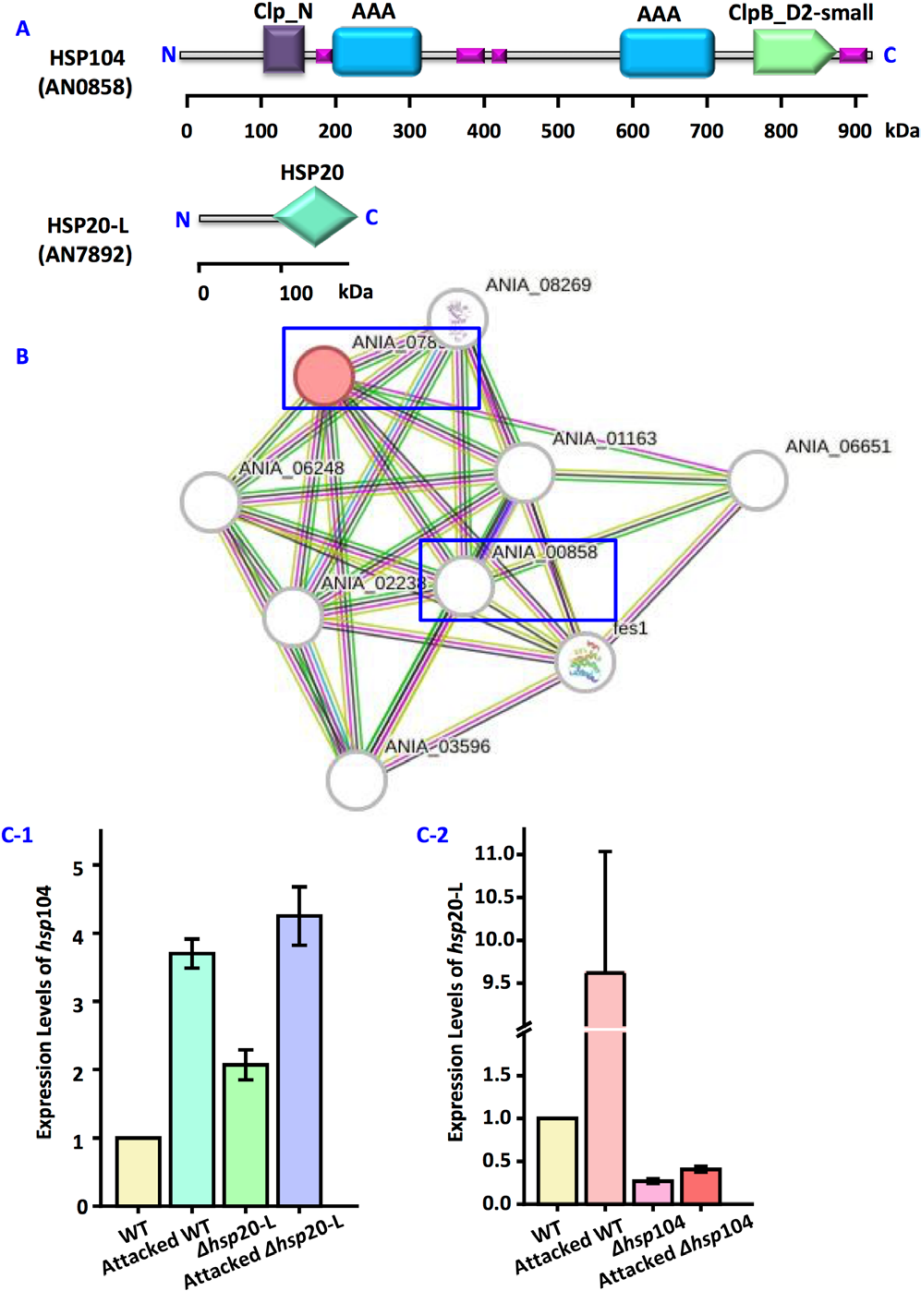
HSP104 and HSP20‐L in *A. nidulans* . (A) Domain structure of HSP104 and HSP20‐L by SMART database (http://smart.embl‐Heidelberg.de) and Protein Blast (http://blast.ncbi.nlm.nih.gov). HSP, heat‐shock proteins; AAA domain, ATPases associated with a variety of cellular activities domain; ClpB_D2‐small domain, C‐terminal, D2‐small domain, of ClpB protein; HSP20, heat‐shock proteins 20. (B) Relationship between HSP104 and HSP20‐L was detected by SMART. (C) Expression levels of *hsp*104 (C‐1) and *hsp*20‐L (C‐2) in different *A. nidulans* strains. Gene expression levels were normalised using the endogenous control gene Actin. Relative mRNA levels were indicated by 2^−ΔΔCT^ method, mRNA levels of *hsp*104 and *hsp*20‐L in the wild‐type strain were set to 1. Values are means ± SD (*n* indicates biological triplicates), and error bars represent SD.

The analysis revealed the upregulation of *hsp*104 (AN0858) and *hsp*20‐L (AN7892) under the *S. curviseta* stress. HSP104 is composed of 927 amino acids. The domain structure using SMART database (http://smart.embl‐Heidelberg.de) and Protein Blast (http://blast.ncbi.nlm.nih.gov) showed that HSP104 harbours heat‐shock proteins (HSP), ATPases associated with a variety of cellular activities (AAA) domain, and ClpB_D2‐small domain, C‐terminal, D2‐small domain, of ClpB protein (Figure 1A). HSP20‐L is composed of 184 amino acids. Domain analysis showed that HSP20‐L only harbours heat‐shock proteins 20 (HSP20) (Figure 1A). In addition, SMART software analyses suggested potential interaction between HSP20‐L and HSP104 (Figure 1B). Figure 1C presents the expression of *hsp*104 and *hsp*20‐L in the wild‐type strain and mutants of *A. nidulans* . Upon *S. curviseta* stress, the wild‐type strain exhibited increased expression levels for both *hsp*104 (3.70 ± 0.212) and *hsp*20‐L (9.62 ± 1.414), compared to control culture conditions. In addition, there was a decrease in the expression of *hsp*20‐L in Δ*hsp*104, while the expression of *hsp*104 in Δ*hsp*20‐L showed an increase. Notably, upon the *S. curviseta* stress, the expression of *hsp*20‐L in Δ*hsp*104 and the expression of *hsp*104 in Δ*hsp*20‐L increased (Figure 1).

### 3.2 *hsp*104 and *hsp*20‐L Are Required for Development

**FIGURE 2:**
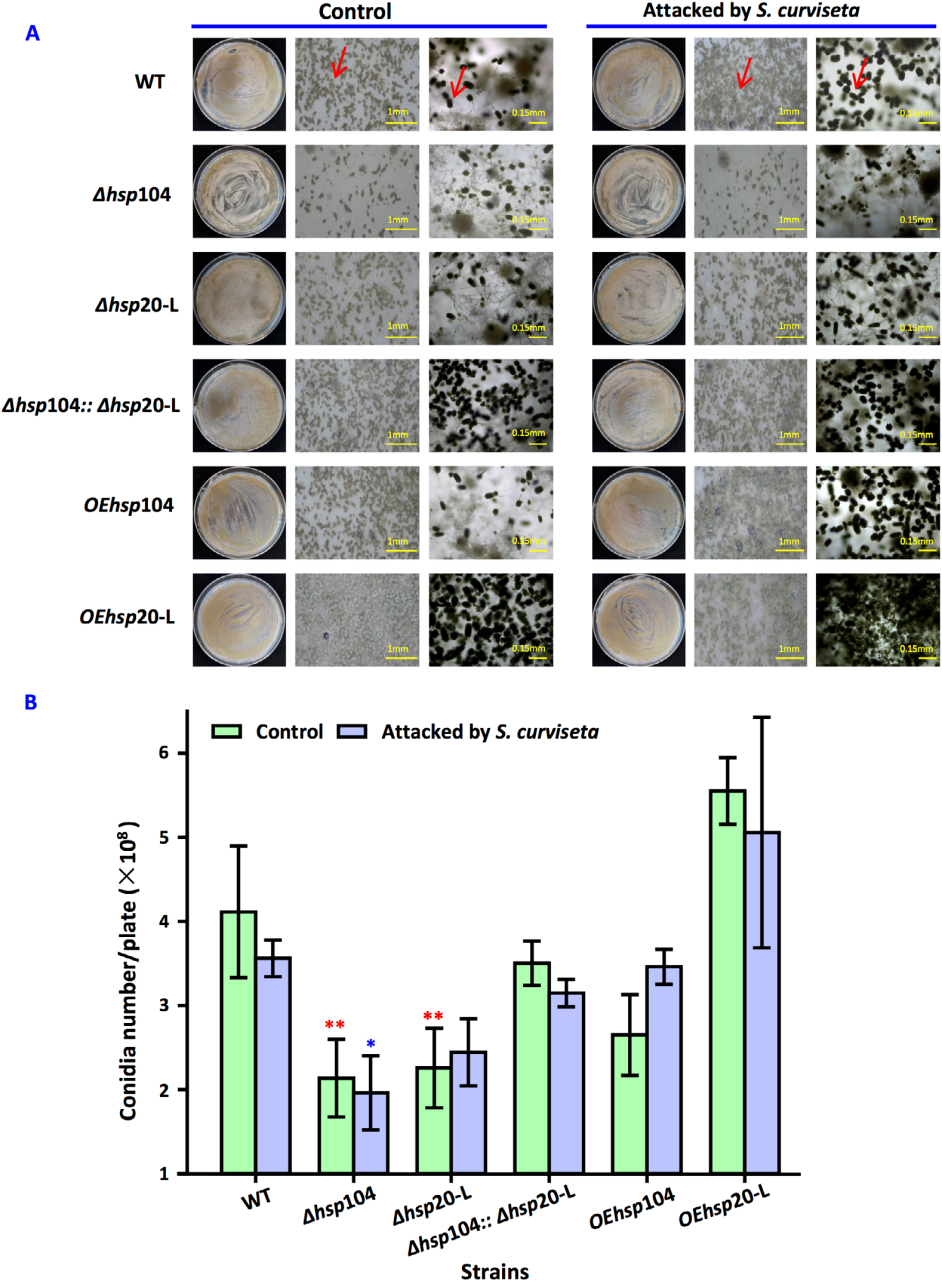
The role of *hsp*104 and *hsp*20‐L in asexual development of *A. nidulans* with and without the attack of *S. curviseta* . (A) Asexual phenotype of the wild type and mutants. Strains were inoculated on GMM plates. (B) Conidia formation of the strains in response to *S. curviseta* . Strains were grown on GMM with and without *S. curviseta* stress. For *S. curviseta* stress, 20 *S. curviseta* were exposed to 72 h cultures of *A. nidulans* . Values are means ± SD (*n* indicates biological triplicates), and error bars represent SD. *p < 0.05, **p < 0.01. The red asterisks represent the difference between mutant and the wild‐type strain in the absence of *S. curviseta* ; the blue asterisks represent the difference between mutant and the wild‐type strain in the presence of *S. curviseta* .

Compared with wild‐type *A. nidulans* , deletion of *hsp*104 or *hsp*20‐L resulted in a reduction of conidia by 48% ± 0.227% and 45% ± 0.024%, respectively. Conversely, the overexpression of *hsp*104 or *hsp*20‐L promoted conidial formation. However, only a modest reduction of 15% ± 0.26% was observed in conidia production from Δ*hsp*104::Δ*hsp*20‐L. Under the *S. curviseta* stress, the strains—including wild‐type strain, Δ*hsp*104, Δ*hsp*104::Δ*hsp*20‐L, and OE*hsp*20‐L exhibited decreased conidia formation. Furthermore, an increase of 8.3% ± 0.088% was noted for Δ*hsp*20‐L under stress conditions and a significant rise of 31% ± 0.350% for OE*hsp*104. The number of conidia detected by haemocytometer is consistent with the image results observed under the microscope (Figure 2).

**FIGURE 3:**
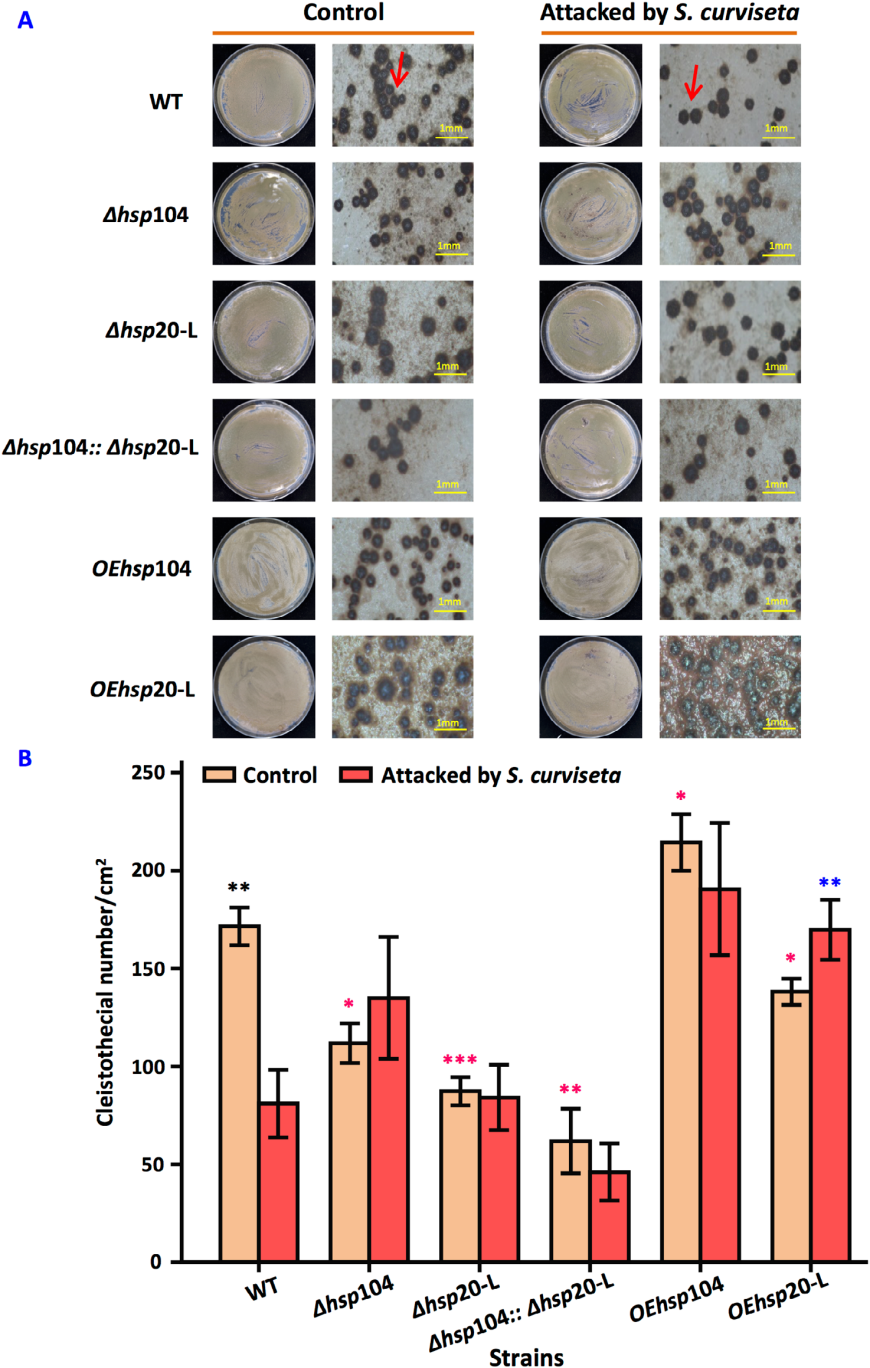
Effect of *hsp*104 and *hsp*20‐L on sexual development of *A. nidulans* with and without the attack of *S. curviseta* . (A) Colony photographs of different strains with and without the attack of *S. curviseta* . (B) Cleistothecial number of the different strains. The wild‐type strains, *hsp* mutants were inoculated on SM. After 72 h of normal incubation, some of the cultures were attacked by 20 *S. curviseta* , while the others were not. Values are means ± SD (*n* indicates biological triplicates), and error bars represent SD. *p < 0.05, **p < 0.01, ***p < 0.001. The red asterisks represent the difference between mutant and the wild‐type strain in the absence of *S. curviseta* ; the blue asterisks represent the difference between mutant and the wild‐type strain in the presence of *S. curviseta* ; the black asterisks represent the difference between the wild‐type, Δ*hsp*104, Δ*hsp*20‐L, ∆*hsp*104::Δ*hsp*20‐L, OE*hsp*104, and OE*hsp*20‐L in the presence and absence of *S. curviseta* .

Compared with the wild‐type strain, the number of cleistothecia was reduced by 35% ± 0.087% and 49% ± 0.015% in Δ*hsp*104 and Δ*hsp*20‐L, respectively. Additionally, there was a notable decrease of 64% ± 0.172% observed in the double mutants. OE*hsp*104 strain showed an increase in cleistothecial formation by approximately 25% ± 0.097% relative to the wild‐type and by about 91% ± 0.354% compared to Δ*hsp*104. In contrast, overexpression of *hsp*20‐L led to an approximate reduction of cleistothecia formation by 19% ± 0.102%, and it did show an increase of around 58% ± 0.257% compared with Δ*hsp*20‐L. The presence of *S. curviseta* stress further enhanced the formation of cleistothecia specifically in OE*hsp*20‐L (Figure 3).

### 3.3 PI Relative Intensity

**FIGURE 4:**
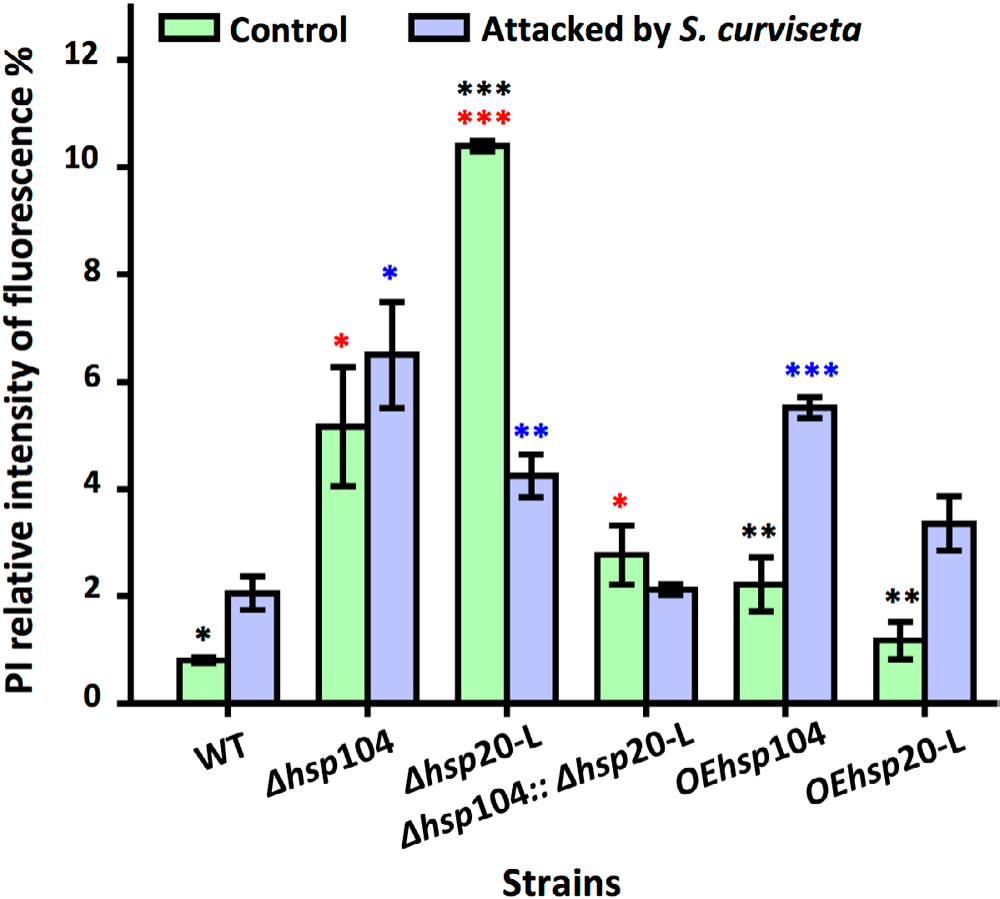
PI relative intensity of fluorescence in wild‐type strain and *hsp* mutants with and without the attack of *S. curviseta* . Values are means ± SD (*n* indicates biological triplicates), and error bars represent SD. *p < 0.05, **p < 0.01, ***p < 0.001. The red asterisks represent the difference between mutant and the wild‐type strain in the absence of *S. curviseta* ; the blue asterisks represent the difference between mutant and the wild‐type strain in the presence of *S. curviseta* ; the black asterisks represent the difference between the wild‐type, Δ*hsp*104, Δ*hsp*20‐L, ∆*hsp*104::Δ*hsp*20‐L, OE*hsp*104, and OE*hsp*20‐L in the presence and absence of *S. curviseta* .

PI fluorescent staining is a technique used to stain DNA with fluorescent dye. PI is a dye that selectively penetrates dead cells while remaining excluded from living cells. In this study, PI staining was employed to detect cellular activity. The fluorescence values were elevated in the mutants compared to the wild‐type strain. The deletion of *hsp*20‐L had the greatest effect on *A. nidulans* , with a fluorescence value of 10.4, approximately 11.9 ± 0.914 times higher than that observed in the wild‐type strain. However, *S. curviseta* stress exacerbated damage to *A. nidulans* , leading to increased fluorescence values in the wild‐type, Δ*hsp*104, OE*hsp*104, and OE*hsp*20‐L (Figure 4).

### 3.4 Content of Chitin

**FIGURE 5:**
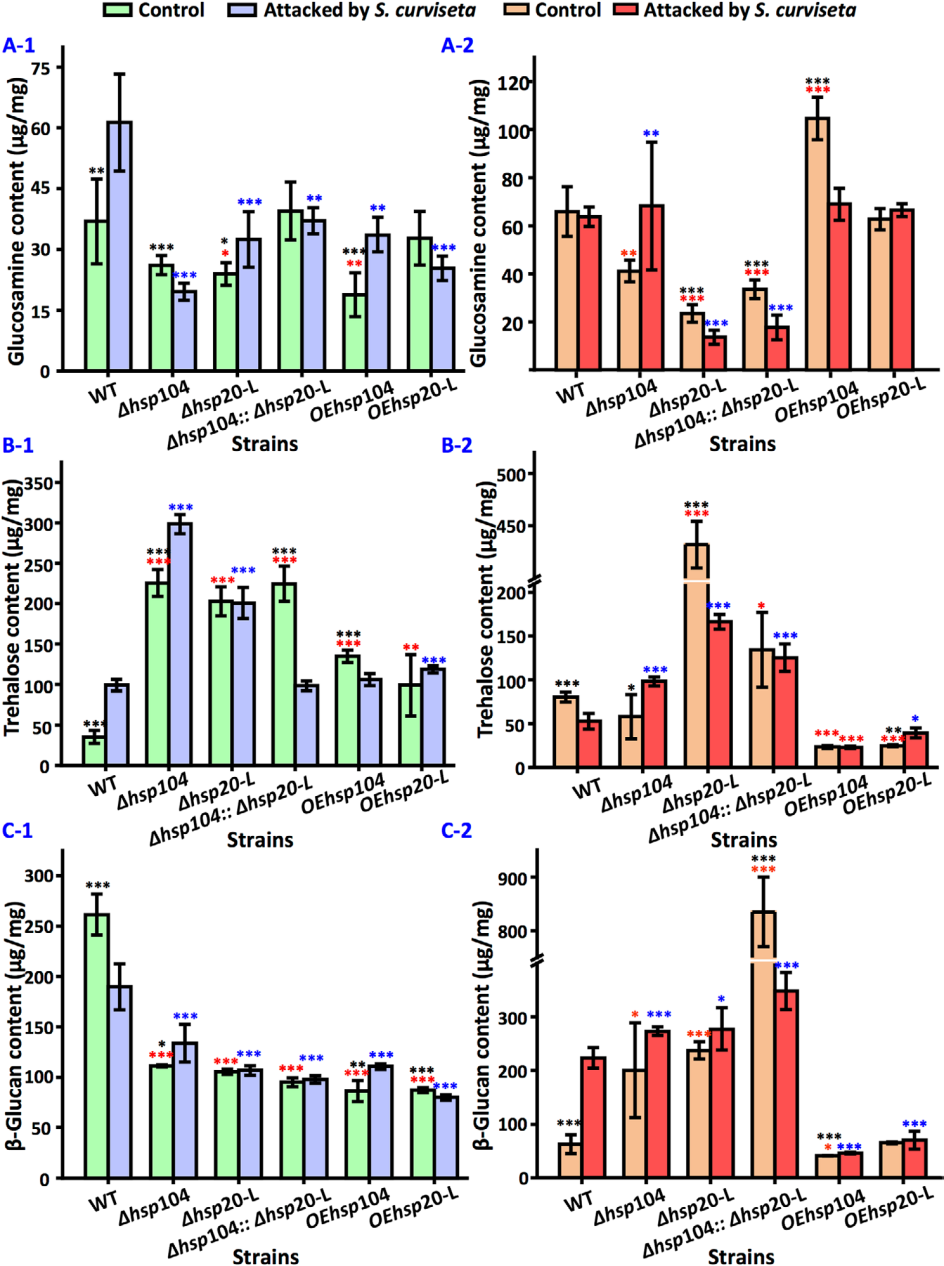
Cell wall components in wild‐type strain and *hsp* mutants with and without attack of *S. curviseta* . (A) Glucosamine accumulation during asexual development (A‐1) and sexual development (A‐2). (B) Trehalose accumulation during asexual development (B‐1) and sexual development (B‐2). (C) β‐glucan content during asexual development (C‐1) and sexual development (C‐2). Values are means ± SD (*n* indicates biological triplicates), and error bars represent SD. *p < 0.05, **p < 0.01, ***p < 0.001. The red asterisks represent the difference between mutant and the wild‐type strain in the absence of *S. curviseta* ; the blue asterisks represent the difference between mutant and the wild‐type strain in the presence of *S. curviseta* ; the black asterisks represent the difference between the wild‐type, Δ*hsp*104, Δ*hsp*20‐L, ∆*hsp*104::Δ*hsp*20‐L, OE*hsp*104, and OE*hsp*20‐L in the presence and absence of *S. curviseta* .

The knockout of either *hsp*104 or *hsp*20‐L led to reduced chitin content in *A. nidulans* at the asexual developmental stage. Compared with the wild‐type strain, the synthesis of chitin was reduced by 29% ± 0.242% and 35% ± 0.205% in Δ*hsp*104 and Δ*hsp*20‐L, respectively. However, only a 7% ± 0.287% increase was noted in Δ*hsp*104::Δ*hsp*20‐L. The overexpression of *hsp*20‐L promoted chitin synthesis, but the overexpression of *hsp*104 did not enhance the amount of chitin. *S. curviseta* stress stimulated an increase in chitin content among the wild‐type strain as well as Δ*hsp*20‐L and OE*hsp*104. Notably, the wild‐type strain exhibited the greatest increase in chitin content at 66% ± 0.727% (Figure 5A‐1).

During sexual development, the deletion of *hsp*104 or *hsp*20‐L resulted in a reduction of chitin content. In addition, the chitin synthesised by Δ*hsp*104::Δ*hsp*20‐L strain was also diminished. Compared to the wild‐type strain, the amount of chitin produced by Δ*hsp*104, Δ*hsp*20‐L, and Δ*hsp*104::Δ*hsp*20‐L decreased by 38% ± 0.036%, 64% ± 0.017%, and 49% ± 0.029%, respectively. Overexpression of *hsp*104 promoted the chitin synthesis, OE*hsp*104 showed a 59% ± 0.197% increase in chitin content compared to the wild‐type strain and a 154% ± 0.503% increase relative to Δ*hsp*104 (Figure 5A‐2).

There was no significant change in the chitin content in wild‐type *A. nidulans* under *S. curviseta* attack. However, there was an increase of 69% ± 0.820% in chitin content within Δ*hsp*104. Meanwhile, OE*hsp*104 demonstrated a 34% ± 0.085% decrease in chitin content upon *S. curviseta* stress. In contrast, Δ*hsp*20‐L showed a 42% ± 0.044% reduction in chitin content under stress, while the chitin content in OE*hsp*20‐L remained relatively stable. Furthermore, Δ*hsp*104::Δ*hsp*20‐L displayed a 47% ± 0.156% decrease in chitin content in response to *S. curviseta* stress (Figure 5A).

### 3.5 Content of Trehalose

At the asexual developmental stage, deletion of *hsp*104 or *hsp*20‐L promoted the accumulation of trehalose. Compared with wild‐type *A. nidulans* , trehalose levels increased by 544% ± 1.237% and 479% ± 0.998% in Δ*hsp*104 and Δ*hsp*20‐L, respectively. Trehalose content in Δ*hsp*104::Δ*hsp*20‐L strain increased by 541% ± 0.997%. Overexpression of *hsp*104 or *hsp*20‐L led to reduced trehalose synthesis, but the levels remained higher than those observed in the wild‐type strain (Figure 5B‐1).

Under *S. curviseta* stress, the trehalose content in the wild type increased by 183% ± 0.802%. The stress also enhanced trehalose levels in Δ*hsp*104, which rose by 32% ± 0.090% compared to the wild‐type strain. However, the strain with overexpression of *hsp*104 did not exhibit an increase in trehalose synthesis upon *S. curviseta* stress. The content in Δ*hsp*20‐L remained essentially unchanged under stress, while OE*hsp*20‐L showed a 20% ± 0.511% increase. Conversely, the trehalose content in Δ*hsp*104::Δ*hsp*20‐L decreased by 56% ± 0.020% upon *S. curviseta* stress (Figure 5B‐1).

During sexual development, there was a reduction of 28% ± 0.362% in trehalose content in Δ*hsp*104, but it increased by 437% ± 0.537% in Δ*hsp*20‐L, and by 67% ± 0.433% in Δ*hsp*104::Δ*hsp*20‐L compared to the wild‐type strain. Overexpression of *hsp*104 and *hsp*20‐L resulted in decreased trehalose accumulation. Under *S. curviseta* stress, only Δ*hsp*104 and OE*hsp*20‐L strains showed an increase in trehalose content (Figure 5B‐2).

### 3.6 Content of β‐Gucan

At the asexual developmental stage, the β‐glucan content was reduced by 57% ± 0.036%, 60% ± 0.033%, and 64% ± 0.040%, respectively, in Δ*hsp*104, Δ*hsp*20‐L, and Δ*hsp*104::Δ*hsp*20‐L compared to the wild type. Overexpression of *hsp*104 and *hsp*20‐L also led to diminished β‐glucan accumulation. The stress of *S. curviseta* inhibited the β‐glucan synthesis in wild‐type and OE*hsp20*‐L strains (Figure 5C‐1).

During the sexual development, deletion of the target gene promoted the synthesis of β‐glucan, especially in the double mutants, which showed an increase in β‐glucan synthesis compared to the wild type. *S. curviseta* stress altered the β‐glucan levels of all the texted strains. Notably, there was nearly a 3‐fold enhancement observed in the wild‐type strain's β‐glucan synthesis. The β‐glucan content decreased in the double mutants, but it was still higher than that in the other strains. *hsp*104 and *hsp*20‐L inhibited the β‐glucan accumulation during sexual development upon stress (Figure 5C‐2).

### 3.7 Activity of SOD

**FIGURE 6:**
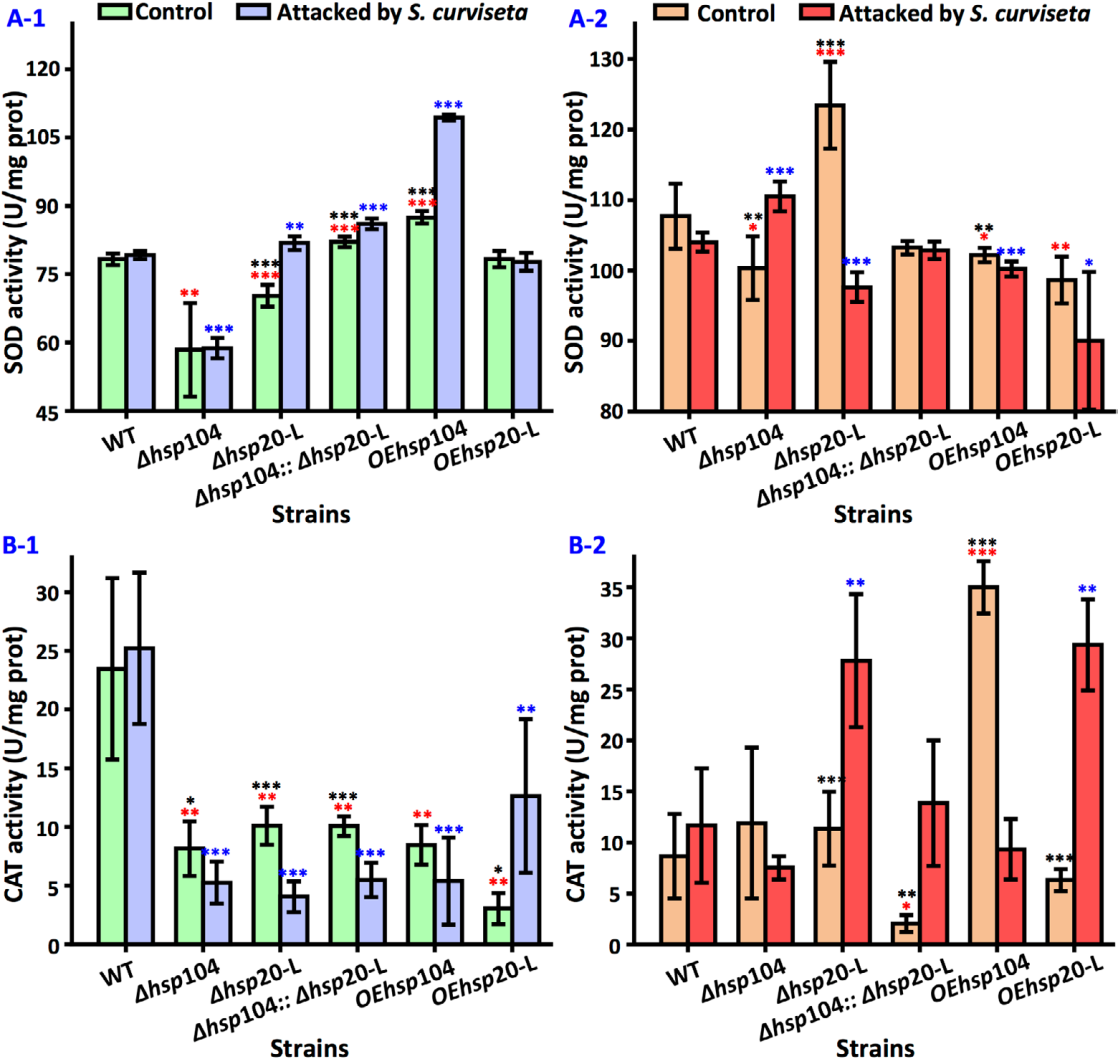
Activity of antioxidant enzyme in wild‐type strain and *hsp* mutants with and without the attack of *S. curviseta* . (A) SOD activity during asexual development (A‐1) and sexual development (A‐2). (B) CAT activity during asexual development (B‐1) and sexual development (B‐2). Values are means ± SD (*n* indicates biological triplicates), and error bars represent SD. *p < 0.05, **p < 0.01, ***p < 0.001. The red asterisks represent the difference between mutant and the wild‐type strain in the absence of *S. curviseta* ; the blue asterisks represent the difference between mutant and the wild‐type strain in the presence of *S. curviseta* ; the black asterisks represent the difference between the wild‐type, Δ*hsp*104, Δ*hsp*20‐L, ∆*hsp*104::Δ*hsp*20‐L, OE*hsp*104, and OE*hsp*20‐L in the presence and absence of *S. curviseta* .

At the asexual developmental stage, the deletion of either *hsp*104 or *hsp*20‐L suppressed SOD activity in *A. nidulans* , but the null of *hsp*104 and *hsp*20‐L genes induced SOD activity. *S. curviseta* stress did not increase SOD activity but induced SOD activity in Δ*hsp*20‐L, Δ*hsp*104::Δ*hsp*20‐L, and OE*hsp*104 (Figure 6A‐1).

At the sexual developmental stage, the knockout of *hsp*104 inhibited SOD activity, whereas the knockout of *hsp*20‐L promoted it. The SOD activity of the other strains was similar as that of the wild‐type strain. Only the activity of SOD in Δ*hsp*104 was induced by *S. curviseta* stress (Figure 6A‐2).

### 3.8 Activity of CAT

During asexual development, CAT activities were reduced in Δ*hsp*104, Δ*hsp*20‐L, and Δ*hsp*104::Δ*hsp*20‐L strains. In addition, overexpression of *hsp*104 or *hsp*20‐L did not enhance CAT activity. *S. curviseta* stress further inhibited CAT activity in Δ*hsp*104, Δ*hsp*20‐L, and Δ*hsp*104::Δ*hsp*20‐L strains (Figure 6B‐1).

At the sexual developmental stage, the double deletion of *hsp*104 and *hsp*20‐L resulted in decreased CAT activity, whereas overexpression of *hsp*104 significantly increased CAT activity. In addition, CAT activity was enhanced in all strains under insect stress except in Δ*hsp*104 and OE*hsp*104 strains (Figure 6B‐2).

### 3.9 Deletion of hsp104 and/or hsp20‐L Affects Metabolites Formation

**FIGURE 7:**
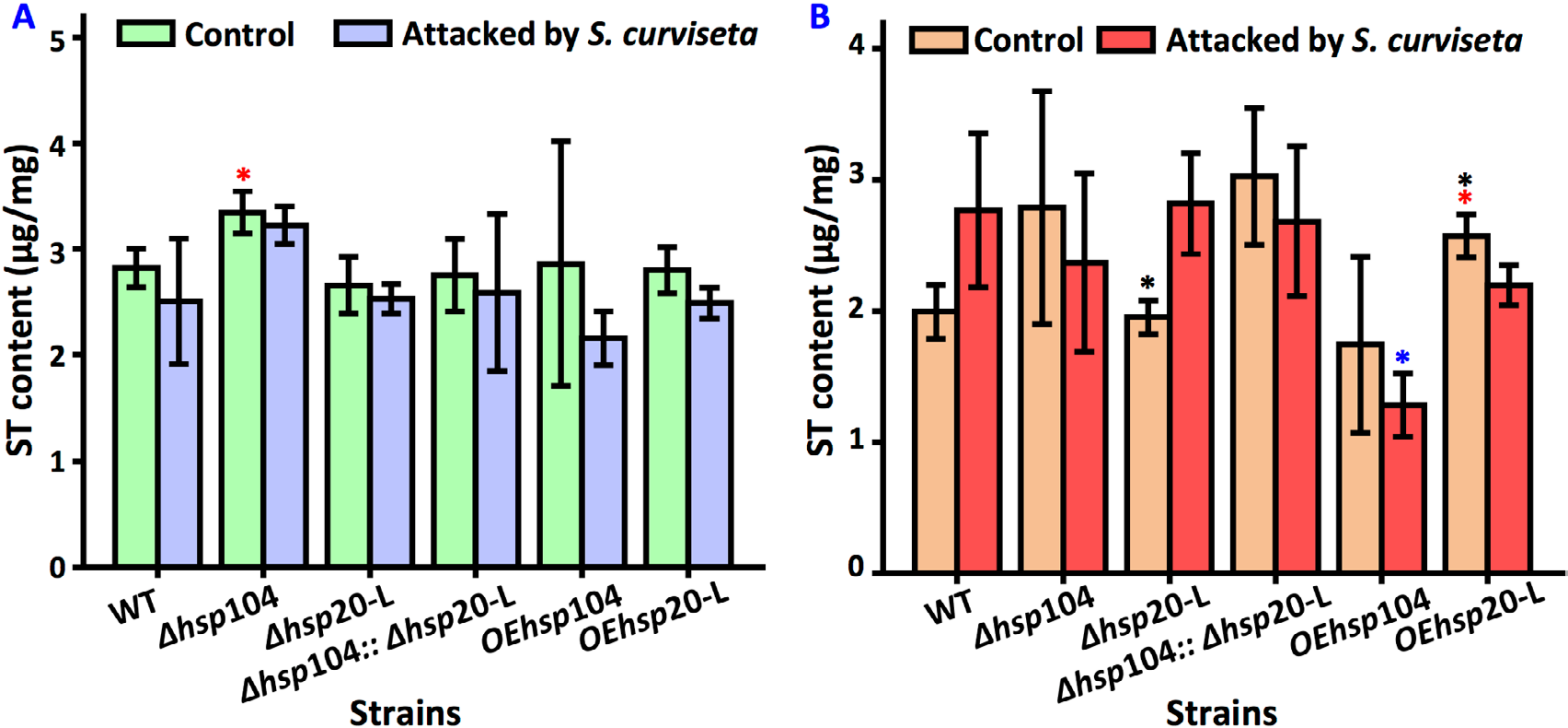
Sterigmatocystin production in wild‐type strain and *hsp* mutants with and without the attack of *S. curviseta* . (A) Amounts of sterigmatocystin in asexual development. (B) Amounts of sterigmatocystin in sexual development. ST, sterigmatocystin. Values are means ± SD (*n* indicates biological triplicates), and error bars represent SD. *p < 0.05. The red asterisks represent the difference between mutant and the wild‐type strain in the absence of *S. curviseta* ; the blue asterisks represent the difference between mutant and the wild‐type strain in the presence of *S. curviseta* ; the black asterisks represent the difference between the wild‐type, Δ*hsp*104, Δ*hsp*20‐L, ∆*hsp*104::Δ*hsp*20‐L, OE*hsp*104, and OE*hsp*20‐L in the presence and absence of *S. curviseta* .

Comparative non‐target metabolomic analysis of *A. nidulans* revealed 2706 detectable metabolites, whereas sterigmatocystin production in the wild‐type strain showed no statistically significant variation with the *S. curviseta* stress, as demonstrated by HPLC (Figure 7). In response to *S. curviseta* stress, 399 metabolites were up‐regulated, mainly IDP, agroclavine, salidroside, megalomicin C1, S‐adenosyl‐1,8‐diamino‐3‐thiooctance, simvastatin, 10beta,14beta‐dihydroxytaxa‐4(20),11‐dien‐5alpha‐yl acetate, 2,3‐dihydrothienamycin, 3‐[4‐(2‐methylbutan‐2‐yl) phenoxy] benzoic acid, and lysyl‐lysine. Additionally, 795 metabolites were down‐regulated, mainly 9(S)‐HpOTrE, roquefortine C, pentahomomethionine, zearalenone, 4‐guanidinobutanal, glutamyl‐glutamine, 2‐aminomuconate semialdehyde, N‐(3‐oxootanoy) homoserine lactone, L‐Ala‐gamma‐D‐Glu‐Dap, and zeatin (Figure S1 and Table S3). The non‐targeted metabolomic data revealed the differential metabolites associated with aflatoxin biosynthesis, galactose metabolism, and biosynthesis of various antibiotics (Figure S2).

The deletion of *hsp*104 led to an increase in sterigmatocystin content compared to that observed in the control strain (Figure 7). In addition, the non‐targeted metabolomic data showed that the knockout of *hsp*104 promoted the synthesis of 363 compounds while inhibiting the synthesis of 622 compounds. Among them, the accumulation of metabolites such as 12‐methyltetradecanoic acid, 3‐butyn‐1‐ol, rifamycin W‐hemiacetal, S‐adenosyl‐1,8‐diamino‐3‐thiooctane, loganate, ethyldihydrocinnamate, 2‐acetyl‐1‐methylpyrrole, DL‐benzylsuccinic acid, 2‐methylmaleate, and 1‐(4‐hydroxyohenyl) ethanol in Δ*hsp*104 were significantly increased (Figure S3 and Table S4).

The deletion of *hsp20*‐L during asexual development did not significantly alter the amount of sterigmatocystin, but increased the synthesis of 493 compounds while decreasing the synthesis of 685 compounds. Among the major enhanced accumulation products were phenazine‐1‐carboxylate, (+)‐sophorol, S‐adenosyl‐1,8‐diamnio‐3‐thiooctane, 2‐(4‐hydroxyphenyl)‐5,6,7,8‐tetrahydroxy‐4H‐1‐benzopyran‐4‐one, 12‐methyltridecanoic acid, desacetylvindoline, L‐fucose‐1‐phosphate, 5,6‐dihydro‐5‐fluorouracil, loganate, and (13S,14R)‐1,13‐dihydroxy‐N‐methylcanadine. Moreover, *hsp*20‐L promoted sterigmatocystin synthesis during sexual development (Figure S4 and Table S5).

The simultaneous deletion of *hsp1*04 and *hsp*20‐L did not affect the level of sterigmatocystin but mainly promoted the accumulation of pantothenol, (+)‐sophorol, aflatrem, 2,3‐dihydrothienamycin, 3‐[4‐(2‐methylbutan‐2‐yl) phenoxy] benzoic acid, N‐(3‐oxooctanoyl) homoserine lactone, mintsulfide, palmitoleic acid, malvidin 3‐O‐glucoside, and megastigmatrienone. The synthesis of 5‐methylcytosine, 4‐hydroxycinnamyl aldehyde, gibberellin A36, D‐glucosamine, 2‐epi‐5‐epi‐valiolone, 2‐methylmaleate, dimethyl‐L‐arginine, 2‐acetyl‐1‐methylpyrrole, glucolesquerellin, and 3‐butyn‐1‐ol was mainly reduced (Figure S5 and Table S6).

## 4 Discussion

The biotic stress is an unfavourable condition for organisms. Hsp70/90 expression in *Bombyx mori* is influenced by microbial challenge (Kausar et al. 2020). *Mr‐hsp*40 and *Mr‐hsp*90 in *Macrobrachium rosenbergii* are involved in cellular stress responses to both temperature and pathogenic bacterial stimuli (Ju‐Ngam et al. 2021).

To overcome the biotic stress, *A. nidulans* upregulated *hsp* expression to perform biological functions. *A. nidulans* responded to *S. curviseta* ‐induced stress through alterations in development, enzyme activity, and cellular composition. HSP104 and HSP20‐L are involved in development and metabolism as well as in stress response in *A. nidulans* . This study demonstrated that both HSP104 and HSP20‐L are required for *A. nidulans* survival in response to *S. curviseta* stress. The mutants were discovered to be sensitive to stress. The content of chitin, trehalose, and β‐glucan indicates that the formation of the fungal cell wall is vital for adaptive response to insect stress. Additionally, *A. nidulans* required enzymes with antioxidative properties to undergo the stress response. *hsp*104 and/or *hsp*20‐L participate in a variety of processes, including biotic stress adaptation, cell wall biosynthesis, sporulation, and enzyme activity. The absence of *hsp*104 or/and *hsp*20‐L leads to the cellular damage of *A. nidulans* . Despite encountering harsh conditions, *A. nidulans* evolved adaptive survival systems.

*A. nidulans* produces conidia during asexual reproduction and cleistothecia containing ascospores during sexual development. These spores endow the fungus with enhanced tolerance and adaptability to adverse environmental conditions. Our results indicate that the knockout of *hsp*104 and/or *hsp*20‐L impaired conidia formation; consequently, Δ*hsp*104 failed to escape predation by producing more conidia upon the attack by *S. curviseta* , but Δ*hsp*20‐L did. Moreover, Δ*hsp*104::Δ*hsp*20‐L formed more conidia. In this context, we hypothesised a complex relationship between *hsp*104 and *hsp*20‐L in regulating the growth and development of *A. nidulans* .

The fungal cell wall serves as the interface between the fungus and its environment. It plays a protective role and is critical for maintaining cellular morphology and integrity. In addition, it is essential for maintaining normal metabolism, ion exchange, and osmotic pressure. The primary components of the fungal cell wall include chitin, deacetyl chitosan, dextran, glucan, and so on. Chitin serves as a crucial structural component of the fungal cell wall (Bulawa 1993). Its biosynthesis is vital for sustaining fungal growth and differentiation. The chitin synthase genes of *A. nidulans* exhibit differential expression in response to developmental status, carbon sources, and environmental stresses (Lee et al. 2004). *A. fumigatus* HSP90 interacts with the key components of the cell wall integrity pathway and plays a collaborative role in heat shock response and adaptation to cell wall stress (Rocha et al. 2021). Our findings indicate that both *hsp*104 and *hsp*20‐L promote the accumulation of chitin, particularly during sexual development. Furthermore, the stress enhanced the chitin content in Δ*hsp*104 during sexual development, and in Δ*hsp*20‐L during asexual development.

During osmotics and challenges related to the cell wall integrity, the trehalose mobilisation proves critical for *A. fumigatus* (Assis et al. 2018). The loss of trehalose within *A. fumigatus* leads to alterations in cell wall structure and integrity (Thammahong et al. 2017). Deletion of *hsp*104 results in increased trehalose accumulation. Conversely, the null of *hsp*20‐L inhibits the accumulation. In addition, the double mutants significantly elevate trehalose levels. Upon the *S. curviseta* ‐induced stress, trehalose content increased in Δ*hsp*104 but decreased in Δ*hsp*20‐L. β‐glucan is a major and essential polysaccharide component of the fungal cell wall (Loza and Doering 2024). The reduction of β‐glucan induced by echinocandins correlates with an increase in levels of chitin, chitosan, and highly polymorphic α‐1,3‐glucans. These compounds physically associate with chitin to maintain cell wall integrity. The compensatory rearrangements in the cell wall assist *A. fumigatus* in tolerating the antifungal effects of drugs (Dickwella Widanage et al. 2024). It was reported that HSP21 is essential for maintaining glucose, trehalose, and glycogen homeostasis under heat shock conditions in *Candida albicans* (Mayer et al. 2013). When exposed to hypersaline environments, *A. sydowii* enhances chitin biosynthesis and incorporates α‐glucan to form thick, stiff, and hydrophobic cell walls. The structural modifications enable the fungus to adapt to hypersaline and salt‐deprived conditions, providing a robust mechanism for withstanding external stress (Fernando et al. 2023).

Fungi generate reactive oxygen species under stress conditions, which can be detrimental to the organism. However, fungi can respond by regulating the antioxidant activity. Our previous studies have demonstrated that the attack of *S. curviseta* triggered bursts of reactive oxygen species (Lu et al. 2024). The accumulation of reactive oxygen species leads to cellular damage and decreased cellular activity. In this study, we observed an increase in PI staining activity in *hsp* mutants, especially upon *S. curviseta* stress, indicating an increase in damage. In response to stress, organisms mobilise antioxidant systems. The activities of enzymes with antioxidant capacities are increased to scavenge free radicals and maintain the balance of free radicals in the cells. In this study, we found that both SOD and CAT activities were reduced in Δ*hsp*104 and Δ*hsp*20‐L during the asexual developmental stage, and *S. curviseta* stress induced SOD activity in all strains, but only CAT activity in wild‐type and OE*hsp*20‐L. Surprisingly, SOD activity was elevated in the double mutants while CAT activity did not decrease compared with the single mutants, whereas CAT activity was significantly reduced in the double mutants during sexual development. It suggests that CAT and SOD play a coordinated role in mediating the antioxidant response while also indicating a complex interplay between the two genes.

Furthermore, *A. nidulans* did not respond to *S. curviseta* stress by significantly increasing sterigmatocystin synthesis, but metabolic yields associated with aflatoxins and antibiotics were increased, suggesting that *A. nidulans* can respond to *S. curviseta* stress through the synthesis of aflatoxin and antibiotics. *A. nidulans* responds to *S. curviseta* stress through an army of compounds in a defence strategy. The metabolic plasticity of fungi has been reported to be coordinated with the survival environment. In fungal‐environmental interactions, resource allocation changes among different defence compounds, depending on the type and duration of stress (Sun et al. 2023; Silva et al. 2020; Zhao et al. 2017).

Taken together, both *hsp*104 and *hsp*20‐L are likely essential genes in response to *S. curviseta* stress in *A. nidulans* . During asexual development, HSP104 and HSP20‐L promoted the conidial formation and the accumulation of chitin and β‐glucan while inhibiting trehalose synthesis. Furthermore, the deletion of *hsp*104 and *hsp*20‐L impaired the activities of SOD and CAT. During sexual development, the deletion of *hsp*104 or/and *hsp*20‐L inhibited the formation of cleistothecia and the accumulation of chitin, but induced the synthesis of β‐glucan. Notably, the deletion of *hsp*20‐L led to increased trehalose levels along with enhanced SOD and CAT activities. Upon insect stress, cellular damage was exacerbated. Specifically, conidial formation and chitin accumulation were not induced in Δ*hsp*104 and Δ*hsp*104::Δ*hsp*20*‐L* mutants during asexual development. Additionally, neither cleistothecia formation nor the content of chitin and trehalose was stimulated by the insect at the sexual stage. Conidiation was reduced by the deletion of *hsp*104, *hsp*20‐L, and even more so when both were deleted. *hsp*104 and *hsp*20‐L were dispensable for trehalose and β‐glucan production, but they were essential for development and chitin biosynthesis. Different strains exhibit distinct strategies to cope with stress. During sexual development, Δ*hsp*104::Δ*hsp*20*‐L* displayed characteristics similar to those observed in Δ*hsp*104 and Δ*hsp*20‐L regarding spores formation as well as chitin and β‐glucan biosynthesis except for its effect on trehalose accumulation. Reports indicate that HSP104 utilises the energy of ATP binding and hydrolysis to rescue stress‐damaged proteins from a previously aggregated state (Lee et al. 2013). HSP20 is an ATP‐independent chaperone that forms oligomeric complexes with client proteins to prevent unfolding and subsequent aggregation (an Ooijen An Ooijen et al. 2010). This study suggested that there may be a feedback regulation mechanism between HSP104 and HSP20‐L. Next, we will conduct an in‐depth study of the interaction between HSP104 and HSP20‐L to reveal the mechanism for counteracting biotic stress in fungi.

## Author Contributions

**Xiaomeng Wang:** methodology, software, data curation, formal analysis. **Juan Xi:** methodology, software, data curation, formal analysis. **Pengxu Chen:** methodology, formal analysis, software, data curation. **Yingying Chen:** methodology, software, data curation. **Keyu Chen:** methodology. **Weifa Zheng:** writing – review and editing, conceptualization, methodology. **Yanxia Zhao:** conceptualization, methodology, software, data curation, formal analysis, writing – review and editing, writing – original draft, funding acquisition.

## Conflicts of Interest

The authors declare no conflicts of interest.

## Supporting information


**Figure S1.** Differential metabolites of *A. nidulans* with and without *S. curviseta* stress. (A) Differential metabolites volcano plot. (B) Differential metabolites histogram.

**Figure S2.** KEGG pathway enrichment scatter plot of differential metabolites.

**Figure S3.** Differential metabolites between Δ*hsp*104 and wild‐type *A. nidulans* . (A) Differential metabolites volcano plot. (B) Differential metabolites histogram.

**Figure S4.** Differential metabolites between Δ*hsp20*‐L and wild‐type *A. nidulans* . (A) Differential metabolites volcano plot. (B) Differential metabolites histogram.

**Figure S5.** Differential metabolites between Δ*hsp*104::Δ*hsp20*‐L and wild‐type *A. nidulans* . (A) Differential metabolites volcano plot. (B) Differential metabolites histogram.

**Table S1.** *Aspergillus* strains used in this study.

**Table S2.** Oligonucleotides used in this study.

**Table S3.** Differential metabolites of *A. nidulans* with and without *S. curviseta* stress.

**Table S4.** Differential metabolites between Δ*hsp*104 and wild‐type *A. nidulans* .

**Table S5.** Differential metabolites between Δ*hsp*20‐L and wild‐type *A. nidulans* .

**Table S6.** Differential metabolites between Δ*hsp*104::Δ*hsp20*‐L and wild‐type *A. nidulans* .

## Data Availability

The data that support the findings of this study are available on request from the corresponding author. The data are not publicly available due to privacy or ethical restrictions.
