# Supplementary figures and images for: HSP104 and HSP20‐L Are Required by Aspergillus nidulans in Response to Attack by Fungivorous Springtail Sinella curviseta
Source: Environ Microbiol Rep. 2025 Jul 6;17(4):e70147. doi: 10.1111/1758-2229.70147 (PMC12229741; doi:10.1111/1758-2229.70147)

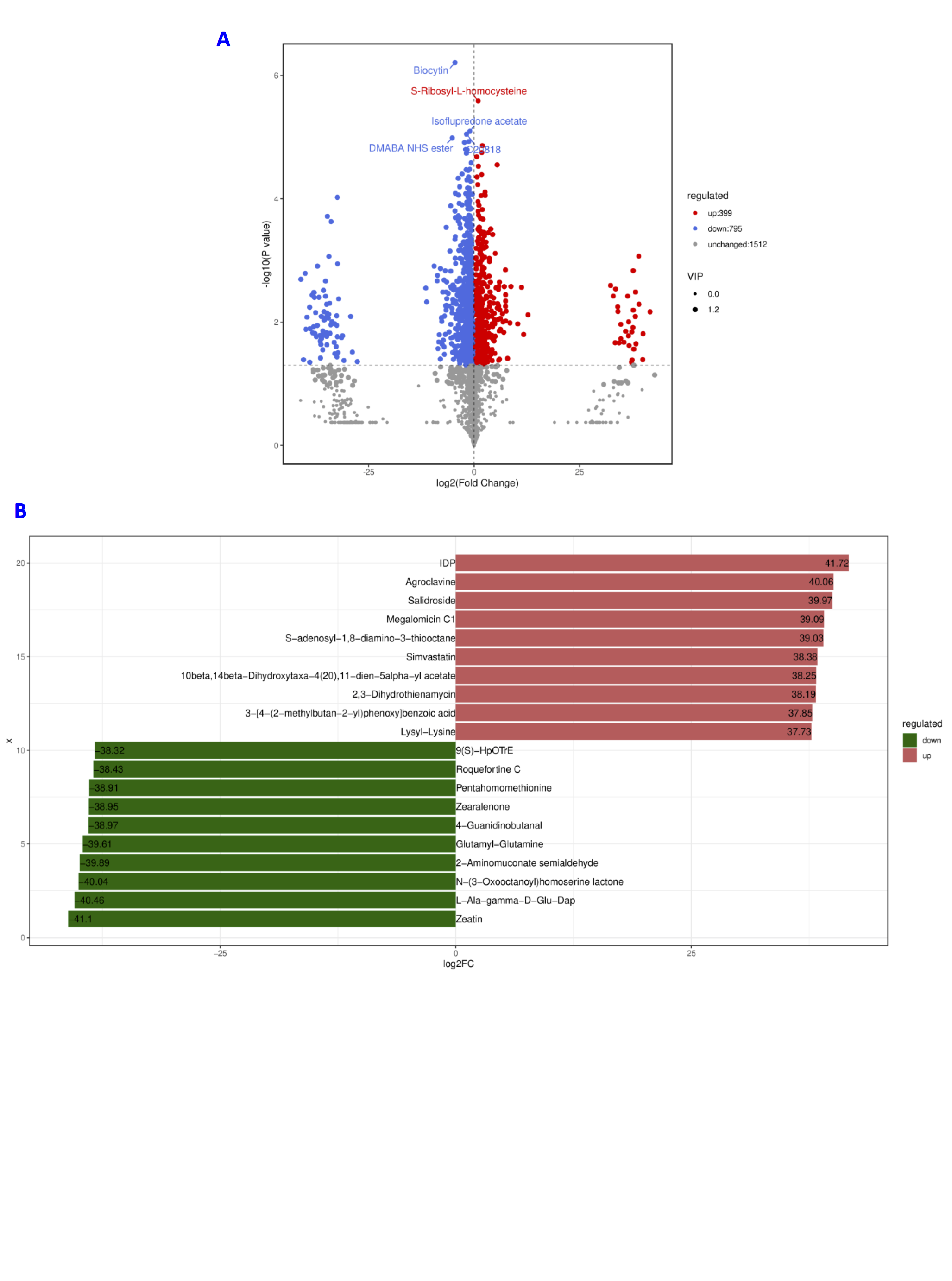

Supplement: Supplementary file 1 — Figure S1. Differential metabolites of A. nidulans with and without S. curviseta stress. (A) Differential metabolites volcano plot. (B) Differential metabolites histogram. [file EMI4-17-e70147-s005.tiff]

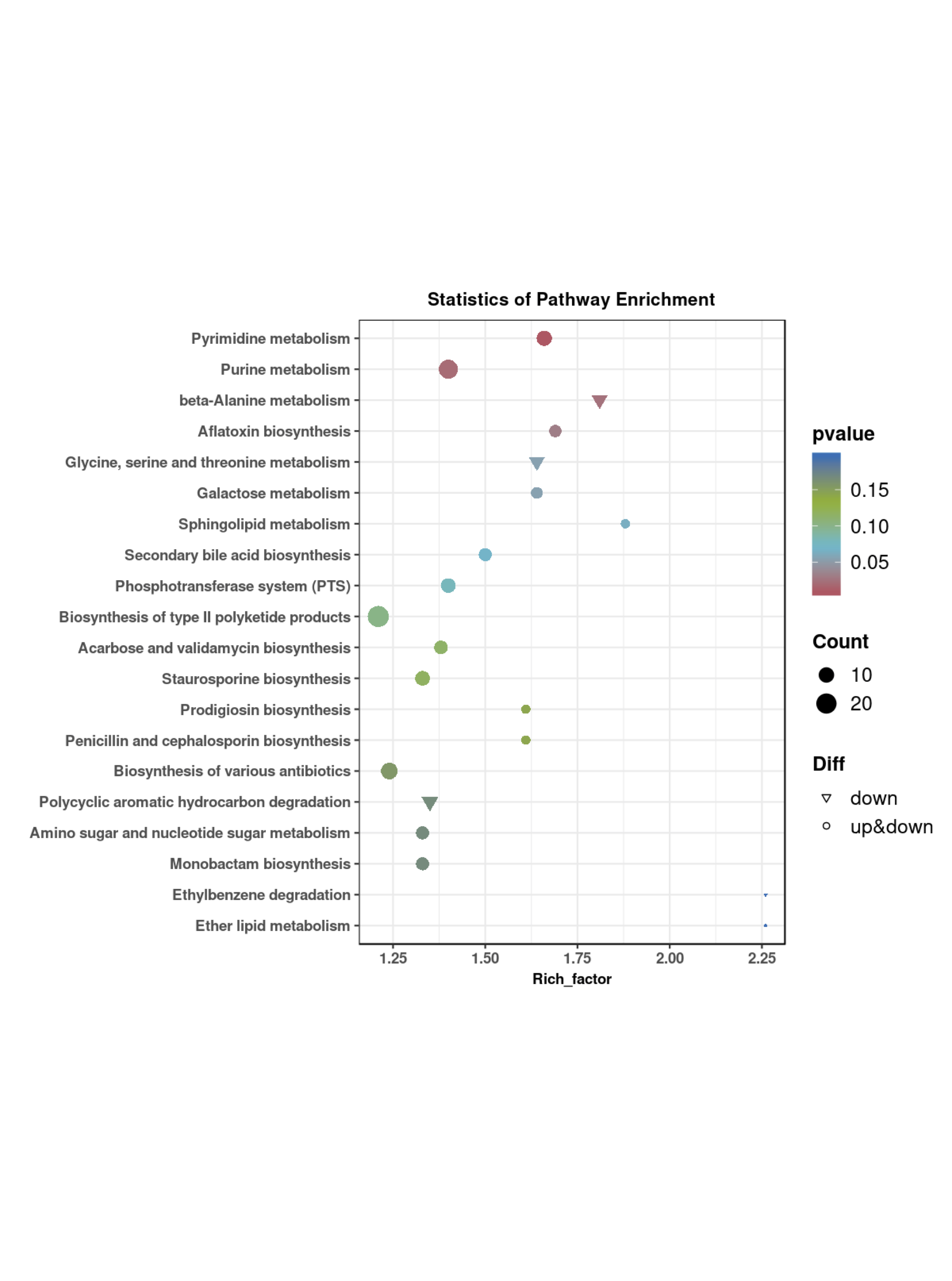

Supplement: Supplementary file 2 — Figure S2. KEGG pathway enrichment scatter plot of differential metabolites. [file EMI4-17-e70147-s011.tiff]

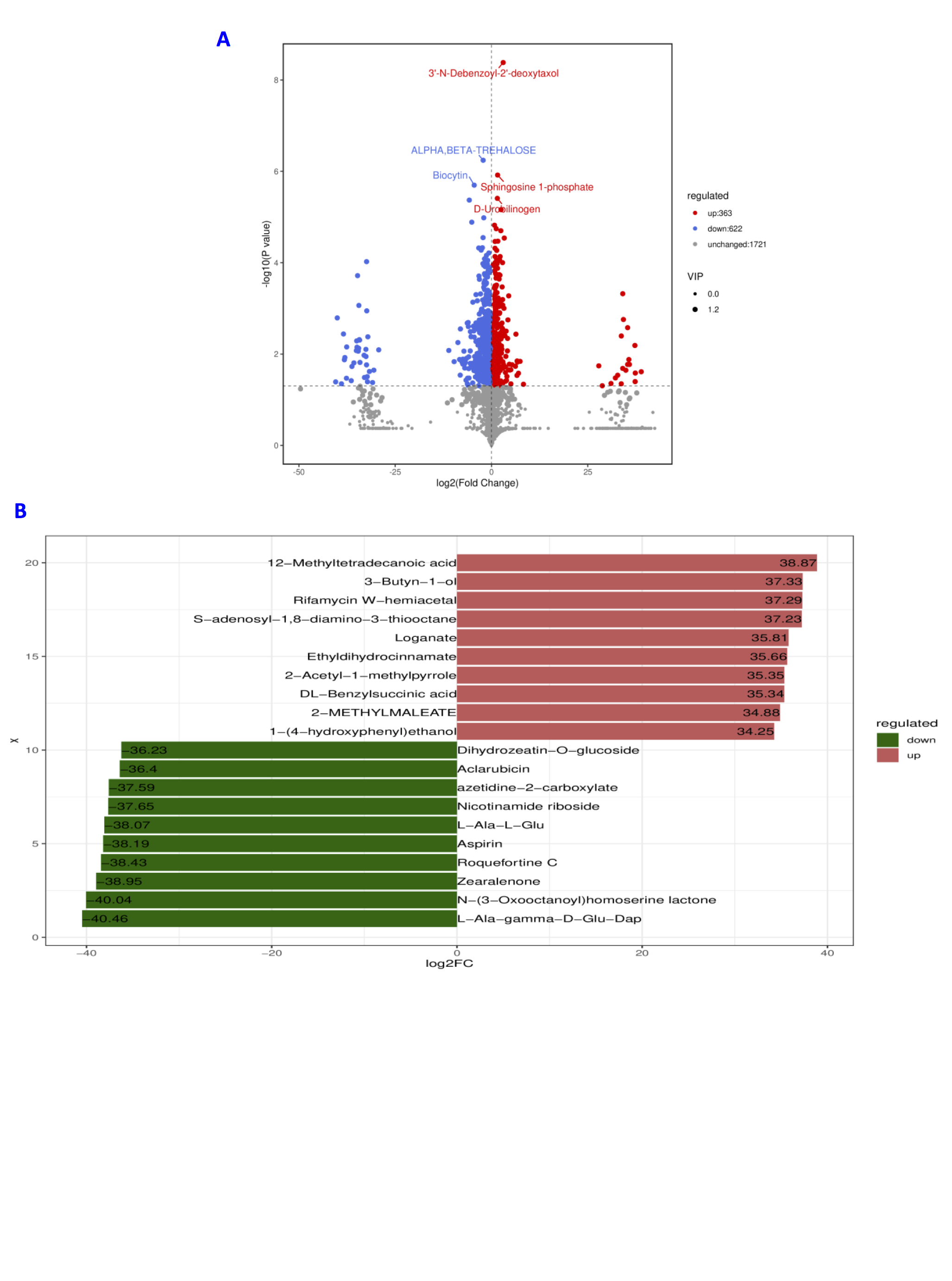

Supplement: Supplementary file 3 — Figure S3. Differential metabolites between Δhsp104 and wild‐type A. nidulans . (A) Differential metabolites volcano plot. (B) Differential metabolites histogram. [file EMI4-17-e70147-s003.tiff]

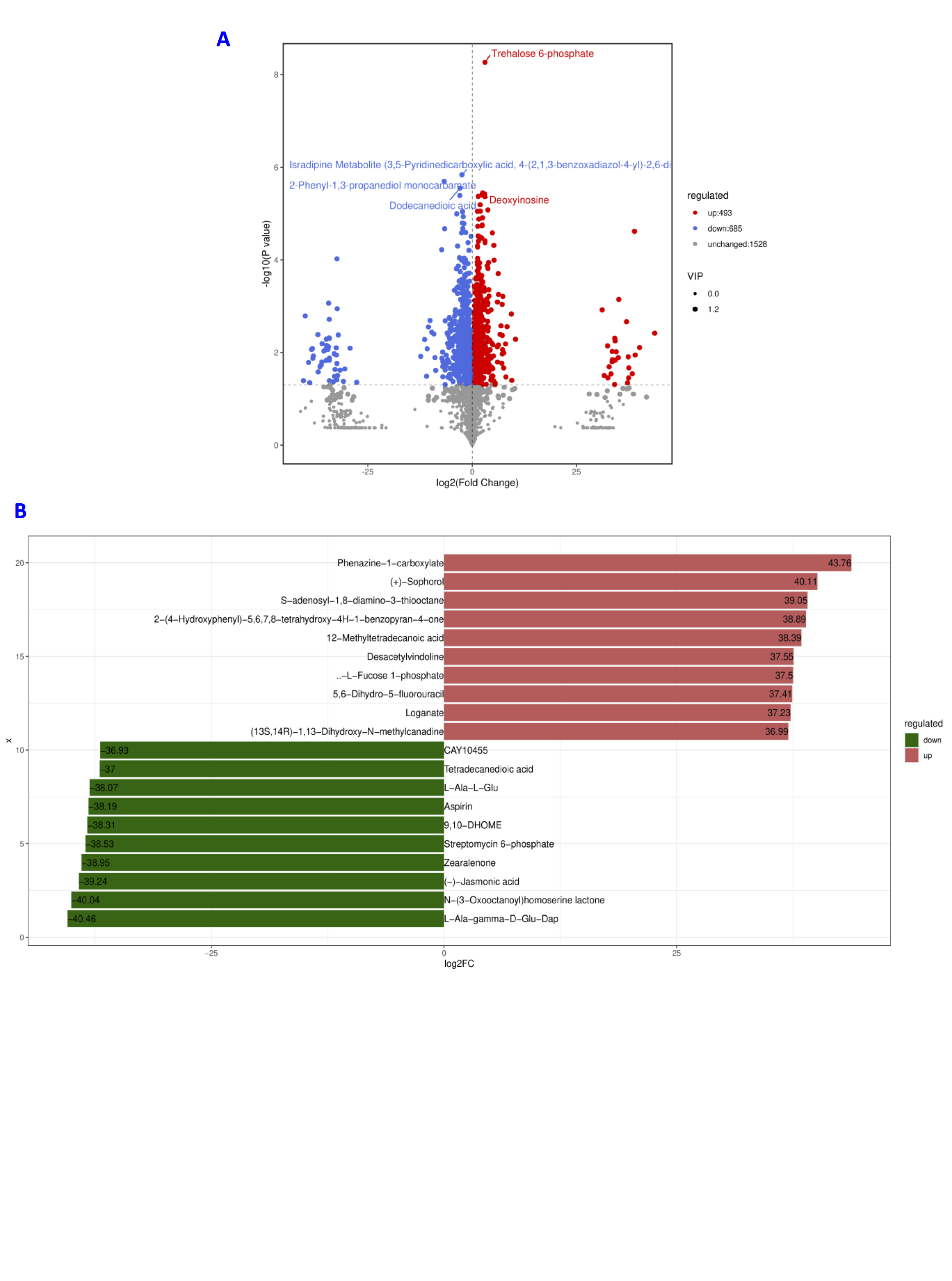

Supplement: Supplementary file 4 — Figure S4. Differential metabolites between Δhsp20‐L and wild‐type A. nidulans . (A) Differential metabolites volcano plot. (B) Differential metabolites histogram. [file EMI4-17-e70147-s007.tiff]

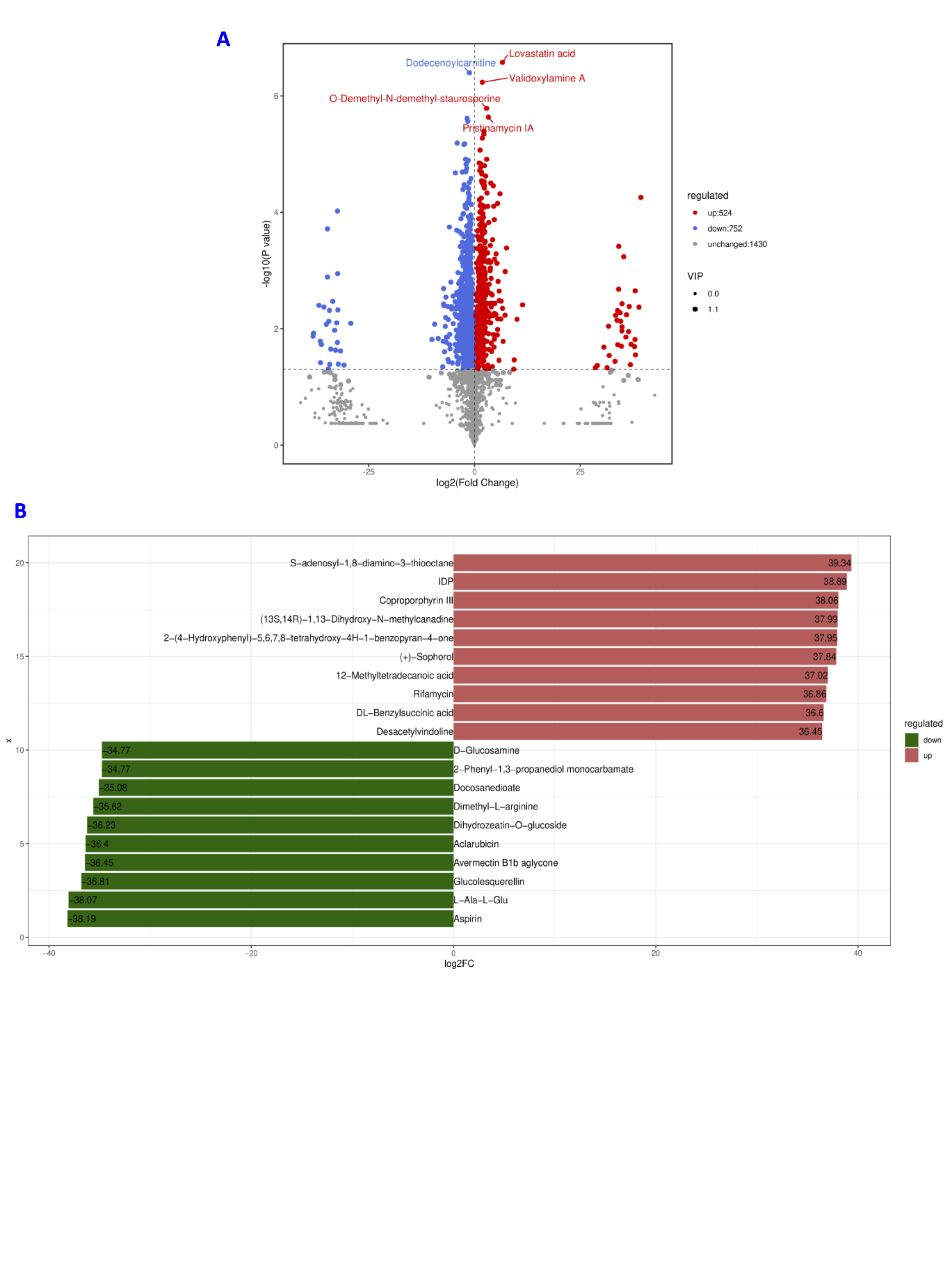

Supplement: Supplementary file 5 — Figure S5. Differential metabolites between Δhsp104::Δhsp20‐L and wild‐type A. nidulans . (A) Differential metabolites volcano plot. (B) Differential metabolites histogram. [file EMI4-17-e70147-s006.tiff]
