# Supplementary material for: HSP104 and HSP20‐L Are Required by Aspergillus nidulans in Response to Attack by Fungivorous Springtail Sinella curviseta
Source: Environ Microbiol Rep. 2025 Jul 6;17(4):e70147. doi: 10.1111/1758-2229.70147 (PMC12229741; doi:10.1111/1758-2229.70147)
Supplement: Supplementary file 6 — Table S1. Aspergillus strains used in this study. [file EMI4-17-e70147-s001.docx]

Table S1. *Aspergillus* strains used in this study

| Strains | Relevant genotype | References |
| --- | --- | --- |
| FGSC A4 | 1. *nidulans* wild type, *veA*^+^ | FGSC ^a^ |
| RJMP 1.59 | *pyrG89*; *pyroA4*; *veA*^+^ | Shaaban et al., 2012 |
| *∆hsp*104 | *pyrG*89; *pyroA*4; △*hsp*104::*AfupyG^+^*;*veA*^+^ | This study |
| *∆hsp*20-L | *pyrG*89; *pyroA*4; △*hsp*20*-L*::*AfupyG^+^*; *veA*^+^ | This study |
| *∆hsp*104*:: ∆hsp*20-L | *pyrG*89; *pyroA*4; △*hsp*20*-L*::*AfupyG^+^*:: *pyroA*; *veA*^+^ | This study |
| OE*hsp*104 | *pyrG*89; *AfupyrG^+^*; *pyroA::nii(p)::hsp*104*::FLAG::pyroA*;*veA^+^* | This study |
| OE*hsp*20*-L* | *pyrG*89; *AfupyrG^+^*; *pyroA::nii(p)::hsp*20*-L::FLAG::pyroA*;*veA^+^* | This study |

^a^Fungal Genetic Stock Center.

**Reference**

Shaaban, M.I.; Bok, J.W.; Lauer, C.; Keller, N.P. Suppressor mutagenesis identifies a velvet complex remediator of *Aspergillus nidulans* secondary metabolism. *Eukaryot Cell*. 2010, 9, 1816-1824.
