# Supplementary material for: HSP104 and HSP20‐L Are Required by Aspergillus nidulans in Response to Attack by Fungivorous Springtail Sinella curviseta
Source: Environ Microbiol Rep. 2025 Jul 6;17(4):e70147. doi: 10.1111/1758-2229.70147 (PMC12229741; doi:10.1111/1758-2229.70147)
Supplement: Supplementary file 7 — Table S2. Oligonucleotides used in this study. [file EMI4-17-e70147-s010.docx]

Table S2. Oligonucleotides used in this study

| Name | Sequence (5’-3’) | Purpose |
| --- | --- | --- |
| AFUpyrG | GCTGAAGTCATGATACAGGCCAAA | pyrG |
| AFUpyrG | ATCGTCGGGAGGTATTGTCGTCAC | pyrG |
| *hsp*104 5F | TCTCCCACTCACGACGGTAT | hsp104_5' flank |
| *hsp*104 5 pyrG R | GGCTTTGGCCTGTATCATGACTTCACGAGGACAGATCCCTTGACC | 5' flank |
| hsp*104* 3 pyrG F | TTTGGTGACGACAATACCTCCCGACGACAAGCTGAGCATAAGCGG | 3' flank |
| *hsp*104 3R | ATGCCCGTTAAGCCACTCAG | 3' flank |
| *hsp*104 nested 5F | AACCGGCCTCGACACTATG | nested 5' flank |
| *hsp*104 nested 3R | CTGTTGCTAACGTGGGGTTG | nested 3' flank |
| *hsp*104 5F | CCTGCGTGCTTCATACTGTG | TEST 5' flank |
| *hsp*104 5R | GGTATTTCAGACCCGCGAAGA | TEST 5' flank |
| *hsp*104 3F | TTGGTGACGACAATACCTCCC | TEST 3' flank |
| *hsp*104 3R | CTGGTGTTTCCACCTTGACG | TEST 3' flank |
| *hsp*20-L 5F | CTGCCTGCTGAACAACGAAC | 5' flank |
| *hsp*20-L 5 pyrG R | GGCTTTGGCCTGTATCATGACTTCAGCGGAAGAGAACGAGCCTAA | 5' flank |
| *hsp*20-L 3 pyrG F | TTTGGTGACGACAATACCTCCCGACGTGGCGTTCCTCTGTCTCATT | 3' flank |
| *hsp*20-L 3R | GTGCTATCTACACCGCAGGC | 3' flank |
| *hsp*20-L nested 5F | CTATTCCTACGAGCCCGACC | nested 5' flank |
| *hsp*20-L nested 3R | TAGAAGAAGCGGATCTGGGGA | nested 3' flank |
| *hsp*20-L 5F | ATTTCAGACCCGCGAAGAGG | TEST 5' flank |
| *hsp*20-L 5R | TCTCTTCGACAAGCAAAGGCT | TEST 5' flank |
| *hsp*20-L 3F | TAACAGCTTGGCATCACGCA | TEST 3' flank |
| *hsp*20-L 3R | TCGGGCCTCAAAAACAGAAGT | TEST 3' flank |
| *hsp*104 5F notI OE | ATAAGAATGCGGCCGCTAAACTATATGAACACCGACAAGTACAC | 5F OE |
| *hsp*104 3R | ATAGTTTAGCGGCCGCATTCTTATTTCGGTACAAATGTATATAT | 3R OE |
| *hsp*20-L 5F notI | ATAAGAATGCGGCCGCTAAACTATATGCCTGAGTACTATCGAGA | 5F OE |
| *hsp*20-L 3R | ATAGTTTAGCGGCCGCATTCTTATATGGAAAATATGTATATTAG | 3R OE |
| pyroA 5F | GCTTCCCCAATGCAATGTCAATACTCCG | 5' flank |
| pyroA 3R | TCAGGGTGTGTATTCAAGTTCGGCG | 3' flank |
| *hsp*104 5F (pyroA) | CCCAGAGCCACCAGCTATC | 5' flank |
| *hsp*104 5R (pyroA) | TCAGAGCTTCTACCGCCTTCCGGAGTATTGACATTGCATTGGGGAAGC | 5' flank |
| *hsp*104 3F (pyroA) | CGCCGAACTTGAATACACACCCTGACGAGGACAGATCCCTTGACC | 3' flank |
| *hsp*104 3R (pyroA) | CGAGTAGTTGCAGAGCGGAT | 3' flank |
| *hsp*104 Nested5F (pyroA) | CCAAGCGAACCAACAAAG | nested 5' flank |
| *hsp*104 Nested 3R (pyroA) | GTCGCCTAACTCCTCTGC | nested 3' flank |
| q*hsp*104 F | GCAGCAGCAGCCTCATCAAT | qPCR F |
| q*hsp*104 R | AACGACGATTCCAGCAAGTG | qPCR R |
| q*hsp*20-L F | AAAGTCTGCGGGTCGGTGAA | qPCR F |
| q*hsp*20-L R | GGACGATTACGACAACCACT | qPCR R |
| q*actin F* | CTCACACTCAGATACCCCAT | actin F |
| q*actin R* | GTCATCTTCTCACGGTTGGA | *actin R* |
